# Generative AI in Game Design: Enhancing Creativity or Constraining Innovation?

**DOI:** 10.3390/jintelligence13060060

**Published:** 2025-05-24

**Authors:** Sultan A. Alharthi

**Affiliations:** College of Computer Science and Engineering, University of Jeddah, Jeddah 21959, Saudi Arabia; saalharthi8@uj.edu.sa

**Keywords:** generative AI, games, game design, user experiece, creativity

## Abstract

Generative AI tools are increasingly being integrated into game design and development workflows, offering new possibilities for creativity, efficiency, and innovation. This paper explores the evolving role of these tools from the perspective of game designers and developers, focusing on the benefits and challenges they present in fostering creativity. Through a mixed-method study, we conducted an online survey (*n* = 42) with game design professionals, followed by in-depth online interviews (*n* = 9), to investigate how generative AI influences the creative process, decision-making, and artistic vision. Our findings reveal that while generative AI accelerates ideation, enhances prototyping, and automates repetitive tasks, it also raises concerns about originality, creative dependency, and undermine of human-authored content. Future work will aim to address these challenges by investigating strategies to balance leveraging AI’s capabilities with preserving the integrity of human creativity. This includes developing collaborative human-AI workflows that maintain human oversight, designing systems that support rather than replace creative decision-making, and establishing ethical guidelines to ensure transparency, accountability, and authorship in AI-assisted content creation. By doing so, we aim to contribute to a more nuanced understanding of generative AI’s role in creative practices and its implications for the game design and development lifecycle.

## 1. Introduction

The intersection of artificial intelligence (AI) and game design has evolved substantially over the past decade, moving from basic AI-driven behaviors toward sophisticated generative models that produce rich and adaptive game content (Hendrikx et al. 2013; Panchanadikar and Freeman 2024; Qiu 2023; Sweetser 2024; Watanabe and Kano 2024; Yannakakis and Togelius 2018). AI in games has traditionally aimed to increase engagement, enhance replayability, and reduce manual workload, but its potential has grown substantially with the advent of generative AI (Hendrikx et al. 2013; Yannakakis and Togelius 2018). Current studies suggest that AI serves not only as an autonomous agent executing pre-written strategies, but also as a creative partner (Medeiros et al. 2025) capable of inspiring designers, suggesting unconventional ideas, and reshaping entire domains, such as writing and narrative design (Sternberg 2024). Early efforts that introduced AI-assisted tools in games such as Tanagra (Smith et al. 2010), the Sentient Sketchbook (Liapis et al. 2013), and Talakat (Khalifa et al. 2018) promised a future in which designers and AI cooperatively build, iterate, and refine game elements. In recent years, with the proliferation of generative AI, this future is becoming a reality with advanced tools such as ChatGPT, Midjourney, and Scenario that have the potential to significantly influence and reshape aspects of game design and development (Chien et al. 2024; Hutson et al. 2024; Liang et al. 2024; Ratican and Hutson 2024; Werning 2024).

However, the remaining challenge lies in balancing creative freedom with developer control. While generative AI can propose a wide range of possibilities, ensuring that these outputs align with the intended design vision requires careful prompt engineering and parameter tuning (Colado et al. 2023; Giray 2023). Researchers are beginning to address these concerns by exploring hybrid approaches that combine human intuition with model-driven creativity (Matthews et al. 2024; Ranella and Eger 2023). Ultimately, the promise of generative AI in game design is not simply more content, but richer and more meaningful content that emerges dynamically from a conversation between human and AI (Chen et al. 2024; Colado et al. 2023). As generative AI tools mature, they open the door to new forms of design and gameplay. Designers can seed the generative process with certain aesthetics or narrative cues, and AI can extrapolate entire worlds, quests, or puzzle structures that feel authentic. In this sense, generative AI is not just a production tool—it may represent an emerging medium of artistic expression in game development (Kim and Kim 2023; Qiu 2023). While generative AI has the potential to significantly streamline content creation and encourage more experimental and innovative design approaches, several critical questions remain unanswered—particularly regarding the perception and adoption of these tools by game designers and their concerns about originality, creative dependency, and human-authored content. Human-authored content refers to creative work produced directly by humans without the assistance of generative AI (Bao and Zeng 2024). These concerns may influence designers’ willingness to adopt such technologies and shape how AI is positioned within creative workflows (Panchanadikar and Freeman 2024; Singh et al. 2024).

Unlike prior research that often focuses on technical implementation or player-facing outcomes, this study contributes a designer-centered perspective by examining how game designers and developers perceive and integrate generative AI tools into their creative workflows. By combining survey data with in-depth interviews, the study captures both broad trends and nuanced, context-specific reflections from a diverse group of designers and developers. This approach offers a grounded understanding of the sociotechnical dynamics shaping generative AI adoption in professional game design practice. To address these gaps, this research examines how game designers and developers engage with generative AI tools, investigating their perceived value, influence on creativity and efficiency, and the obstacles that hinder their effective integration. Specifically, this study seeks to answer the following research questions:

- RQ1: how do game designers and developers perceive the value and potential of generative AI tools in their workflows?
- RQ2: how do generative AI tools influence creativity, productivity, and efficiency in game design and development?
- RQ3: what are the primary challenges and concerns game designers and developers face when using generative AI?

This study follows an exploratory research design, aiming to surface nuanced insights from designers and developers rather than test predefined hypotheses. By centering open-ended research questions, the study allows for emergent themes to arise from the data, reflecting the complexity of integrating generative AI into creative practice. Through a mixed-methods approach, we explore the impact of generative AI tools on creativity and innovation in game design and development. By investigating our research questions, the study aims to provide a clearer understanding of the human–AI co-creative process, offering insights that can guide the development of more intuitive, transparent, and impactful generative AI tools for the future of game design and development. Our findings reveal that, while generative AI significantly accelerates ideation, enhances the prototyping process, and automates repetitive or labor-intensive tasks, it also introduces notable concerns that warrant deeper exploration. Specifically, questions surrounding the originality of AI-generated outputs remain prominent, as the reliance on training data may result in derivative or uninspired creations.

The remainder of this paper is structured as follows. Section 2 reviews relevant literature on generative AI in game design and creative practice. Section 3 outlines the research methodology, including data collection and analysis procedures. Section 4 presents the findings from both the survey and interviews. Section 5 discusses the implications of these findings, and Section 6 outlines the study’s limitations. Finally, Section 7 offers concluding remarks and directions for future research.

## 2. Background

Generative artificial intelligence (generative AI) has rapidly transformed the field of game design and development, influencing a wide range of creative and technical processes. AI technologies such as procedural content generation, AI-driven narrative engines, and automated asset creation have introduced both new opportunities and significant challenges for game designers and developers (Ratican and Hutson 2024). The integration of AI in game design workflows has led to the emergence of novel game mechanics and interactive storytelling methods, redefining the role of human creators in the gaming industry (Li 2024; Sun et al. 2023). As generative AI becomes more prevalent, its reception among game designers and developers varies. Some view AI as an essential tool that enhances creativity and streamlines production, while others raise concerns about artistic authenticity, creativity, and ethical implications (Panchanadikar and Freeman 2024). In particular, indie game developers have expressed mixed feelings regarding the use of AI, with some seeing it as a democratizing force that enables solo developers to achieve ambitious projects, whereas others fear that AI-generated content could lead to homogenization in game design (Panchanadikar and Freeman 2024; Panchanadikar et al. 2024). This work explores the multifaceted role of generative AI in game creation and examines how designers and developers perceive its growing influence.

### 2.1. The Role of Generative AI in Game Design and Development

Generative AI plays an increasingly central role in various aspects of game development, from concept art creation to procedural world-building and adaptive storytelling. AI-powered tools such as Stable Diffusion, Midjourney, and ChatGPT have made it possible to generate high-quality game assets, reducing the time and labor required for traditional design processes (Begemann and Hutson 2024). AI-driven game engines are also being used to create adaptive gameplay experiences, allowing for more dynamic and player-driven narratives (Boucher et al. 2023; Shan and Michel 2024). A significant application of AI in game design is procedural content generation, where AI algorithms generate game environments, characters, and items in real time. This approach has been successfully implemented in games like No Man’s Sky, which utilizes AI-generated planetary landscapes to create an expansive universe with billions of procedurally generated worlds (Ratican and Hutson 2024). AI has also enabled the automation of game playtesting and quality assurance, with tools being used to identify and resolve gameplay issues. However, concerns have been raised about the creative limitations of AI-generated content. While AI can generate large volumes of assets quickly, some game designers argue that these assets often lack the artistic depth and intentionality of human-created designs (Lee et al. 2023). Additionally, AI-generated level designs and narrative structures may struggle to maintain thematic coherence, leading to inconsistencies in game worlds (Begemann and Hutson 2024).

Beyond procedural systems, AAA studios are now integrating AI tools into narrative design workflows. Ubisoft’s research division, La Forge, for instance, has developed Ghostwriter, a generative AI tool that assists narrative designers by producing first-draft NPC dialogue (Ubisoft La Forge 2024). While human writers remain central to content refinement, the tool enables faster prototyping of branching interactions and routine in-game conversations, illustrating how AI can enhance creative efficiency without fully replacing human input. Sweetser (2024) conducted a preliminary scoping review focusing on AI and video games. Their review identified key focus areas in game AI, game development, narrative, and game research, providing an early state of the field and laying the groundwork for future research. On the other hand, Panchanadikar and Freeman (2024) provide critical insights into the evolving role of generative AI within indie game development. Through an analysis of online posts and comments, the authors highlight both opportunities and challenges that generative AI presents to indie developers. They emphasize that, while these tools can democratize game production by reducing costs, streamlining workflows, and enhancing creativity, they also introduce risks related to intellectual property, career stability, and the coherence of artistic outputs. They advocate for a balanced integration of generative AI, proposing design principles that preserve human creativity, autonomy, and ethical practices, thus fostering a sustainable and inclusive game development ecosystem.

### 2.2. Techniques Used in Generative AI Tools for Game Design and Development

AI has long played a pivotal role in video games, influencing everything from non-player character (NPC) behavior to procedural content generation. At its core, AI in games focuses on creating systems that can adaptively respond to player input, exhibit human-like decision-making, and contribute to more engaging play experiences. Yannakakis and Togelius (2018) delve into the foundations of AI in games, highlighting how techniques such as evolutionary computation, reinforcement learning, and behavior trees have shaped the landscape of video games. The application of generative AI in game design has gained momentum as researchers and practitioners seek to push beyond static, handcrafted experiences (Boucher et al. 2023; Chien et al. 2024; Panchanadikar et al. 2024). Generative AI, often realized through machine learning models capable of producing a wide variety of content, encourages a more fluid and iterative design process (Yannakakis and Togelius 2018). Recent advances in generative AI have enabled the dynamic creation of game content using algorithmic techniques. These techniques employ a variety of machine learning models and algorithms, most notably procedural content generation (PCG), Generative Adversarial Networks (GANs), and transformer-based language models such as GPT (Colado et al. 2023; Maleki and Zhao 2024; Werning 2024). Procedural content generation (PCG) refers to algorithmic systems designed to produce game content such as levels, maps, or quests automatically. Early PCG models used rule-based systems, but current approaches often utilize algorithms and machine learning to generate complex, adaptive content dynamically (Maleki and Zhao 2024). On the other hand, Generative Adversarial Networks (GANs) are widely used for producing visual game assets. By training two competing neural networks—a generator and a discriminator—GANs can create lifelike images and textures that blend seamlessly with hand-crafted game environments. GANs have also been used in game ideation and prototyping processes, offering designers high-fidelity mockups with minimal manual input (Werning 2024). More recently, transformer-based large language models (LLMs) such as GPT-3 have enabled automatic script writing, dynamic dialogue systems, and interactive narrative branching in games. These models can co-create with developers by generating code snippets, quests, and character bios in natural language, streamlining game production workflows (Colado et al. 2023).

### 2.3. Perceptions of Game Designers and Developers on AI Integration

The perception of AI among game designers and developers is highly nuanced, with both optimism and skepticism shaping discussions on its role in creative workflows (Boucher et al. 2024; Ersoy 2024). One of the primary benefits of AI integration is its ability to enhance efficiency and reduce the workload on designers. AI-generated concept art, for instance, allows designers to rapidly explore multiple visual styles before committing to a final design, thereby accelerating the conceptualization phase of game development (Panchanadikar and Freeman 2024). AI has also been seen as a valuable tool for indie developers who lack the financial resources to hire large teams. AI-powered engines can generate code, textures, animations, and sound assets, enabling solo developers to create complex games that would otherwise require extensive manpower (Panchanadikar et al. 2024). However, indie developers also express concerns about the over-reliance on AI tools, fearing that it could lead to a decline in the uniqueness and craftsmanship that characterize many indie games (Panchanadikar and Freeman 2024). Intellectual property and ethical concerns surrounding AI-generated content have also been widely discussed (Singh et al. 2024). Many developers worry about the legal and ethical implications of AI models that are trained on existing artwork without explicit permission from original creators (Boucher et al. 2023; Hutson et al. 2024; Lee et al. 2023).

### 2.4. AI in Game Storytelling and Narrative Design

Beyond visual and technical aspects, AI is also being integrated into game storytelling, with AI-driven narrative engines generating dynamic and adaptive game narratives (Vinchon et al. 2024). Games such as AI Dungeon and 1001 Nights utilize large language models to create interactive storytelling experiences where players’ choices influence the unfolding narrative (Sun et al. 2023). This approach allows for greater player agency, as AI can generate personalized storylines based on user input. Despite these innovations, AI-driven storytelling presents significant challenges. AI-generated narratives often lack the emotional depth and character development found in human-written stories (Choi 2024). Additionally, AI-generated dialogue systems may struggle with maintaining coherence over long story arcs, leading to inconsistencies in character behavior and plot progression (Boucher et al. 2023). Some developers have noted that, while AI can assist in generating story ideas, human writers are still needed to refine and structure narratives in a way that resonates with players emotionally (Panchanadikar et al. 2024).

Another area where AI is transforming storytelling is in the creation of procedural dialogue and NPC interactions. AI-powered NPCs can dynamically respond to player actions, creating a more immersive and responsive game world (Panchanadikar and Freeman 2024). However, concerns remain about the unpredictability of AI-driven characters, as AI-generated dialogues can sometimes result in awkward or nonsensical interactions (Choi 2024). As AI technology continues to evolve, future research should focus on developing frameworks for responsible AI integration in game development. Ethical guidelines and industry standards must be established to ensure fair use of AI-generated content while maintaining human creativity at the forefront of game design. The challenge moving forward will be to find a balance between leveraging AI’s capabilities and preserving the artistic essence that defines the gaming industry.

As AI continues to shape the gaming industry, its impact is also being felt in education and training for game developers (Arnecke et al. 2024; Gao et al. 2024a, 2024b; Whitaker et al. 2024). Several institutions have begun incorporating AI-based tools into game design curricula, providing students with access to AI-driven asset generation (Choi 2024; Li et al. 2024; Pérez-Colado et al. 2024). AI is also being used in game testing education, with AI-driven assistants helping students design playtesting protocols and analyze player feedback (Amresh 2023; Muengsan and Chatwattana 2024; Wang 2024). However, there is still some skepticism about the role of AI in game design education. Some educators argue that an over-reliance on AI tools may hinder students’ ability to develop foundational skills in traditional game design methods (Hutson et al. 2024; Sun and Cho 2024). Others suggest that AI should be viewed as an augmentative tool rather than a replacement for human creativity, with curricula emphasizing a hybrid approach that balances AI-generated content with human artistic input (Lee et al. 2023).

### 2.5. Summary

The integration of generative AI in game design and development presents both exciting opportunities and significant challenges, yet much of the current discourse has been shaped by theoretical analyses or data collected through online forms, with limited direct engagement with the practitioners at the forefront of this technological shift. While AI has the potential to enhance efficiency, streamline workflows, and unlock new creative possibilities, concerns about artistic authenticity, ethical dilemmas, and the balance between human and AI contributions remain significant. These concerns are amplified by the lack of in-depth studies that actively involve game designers and developers in meaningful dialogue to capture their nuanced perspectives. Unlike many prior studies that rely on secondary data or only qualitative data (Boucher et al. 2024; Panchanadikar and Freeman 2024; Panchanadikar et al. 2024), this research directly engages with game designers and developers through a mixed-method study, offering a richer, more grounded understanding of how generative AI is reshaping the creative process and professional workflows. By centering the voices of designers and developers, this study highlights both their enthusiasm for the transformative potential of generative AI and their apprehensions regarding its impact on originality and the human-driven artistry that defines game design.

## 3. Methodology

This study employed a mixed-methods approach to explore the impact of generative AI tools on creativity and innovation in game design. All procedures involving human participants complied with the University of Jeddah research committee ethical standards and with the 1964 Helsinki Declaration and its later amendments or comparable ethical standards. Written informed consent was obtained from all subjects involved in the study.

### 3.1. Online Survey

**Figure 1:**
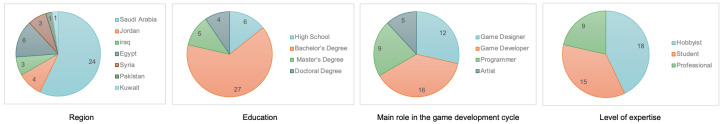
Participant demographics: region, education, role, and expertise level.

To answer our research questions, we conducted an online survey with game designers (*n* = 42). Participants were recruited from a game design and development social media community in WhatsApp (Figure 1). This community was selected due to its focus on professional and semi-professional game developers, and its high level of activity related to emerging technologies in game design. While this recruitment method enabled access to informed and engaged professionals, it may reflect a subset of voices with shared exposure to game design and AI-related discourse. Participants who completed the survey ranged in age from 19 to 45 years old (*M* = 31, *SD* = 5.72). Of the participants, 66% identified as male, while 34% identified as female. The survey developed for this study was informed by the Technology Acceptance Model (TAM) (Davis et al. 1989) and the Unified Theory of Acceptance and Use of Technology (UTAUT) (Venkatesh et al. 2012). The survey included both closed and open-ended items addressing usefulness, ease of integration, creativity enhancement, and ethical concerns. A mix of Likert-scale and free-text responses were used. Both TAM and UTAUT frameworks provided a robust foundation for understanding the adoption and integration of generative AI tools in game design. TAM focuses on perceived usefulness and ease of use as key determinants of technology adoption, which were reflected in survey items assessing productivity, creativity enhancement, and workflow integration. Similarly, UTAUT emphasizes factors such as performance expectancy, effort expectancy, social influence, and facilitating conditions, all of which were incorporated to examine how game designers perceive and adopt generative AI into their work.

### 3.2. Online Interviews

Following the survey, we conducted 9 one-on-one online interviews with game designers to gain a deeper understanding of their experiences and challenges with generative AI in game design. Interviews followed a semi-structured guide covering experiences with generative AI, specific use cases, perceived benefits, creative limitations, and ethical reflections. The interview participants were recruited directly through the initial survey. They ranged in age from 27 to 42 years old (*M* = 36, *SD* = 4.98) and 7 identified as male, while 2 identified as female. These interviews provided an opportunity to explore individual perspectives in greater detail, capturing nuanced accounts of how such tools accelerate ideation, enhance the prototyping process, and automate repetitive tasks.

While the sample size for this study is modest (42 survey participants and 9 interviewees), it was purposefully selected to capture a diverse cross-section of professionals across roles and expert types. This method aligns with the study’s exploratory objective, which prioritized rich, contextual insights over statistical generalization.

### 3.3. Data Analysis

Data from the surveys and interviews were analyzed to identify the perception and main challenges faced by game designers in adopting generative AI tools. After all the data were collected, they were imported into ATLAS.ti, a qualitative data analysis software, to facilitate systematic organization and coding. The main author conducted an exploratory, thematic analysis approach (Braun and Clarke 2012) was employed to systematically identify recurring themes and patterns across the data, ensuring a rigorous and consistent analysis process. The analysis followed a structured multi-step process:

- First, we familiarized ourselves with the data by thoroughly reading survey responses, interview transcripts, and observation notes.
- Next, we generated initial codes by systematically categorizing meaningful data excerpts, capturing recurring issues such as *ownership barriers*, *ethical concerns*, and *creative dependency*.
- After coding, we identified preliminary themes by grouping related codes into broader categories that reflected patterns in the data.
- These themes were then reviewed and refined, ensuring that they accurately represented the data and were distinct from one another. Examples of emergent themes included *automation*, *low-fidelity prototype*, and *ideation*.
- Finally, the refined themes were analyzed in depth and synthesized into a coherent narrative to report the findings. This approach ensured that the analysis captured the complexity of participants’ experiences while providing actionable insights into the barriers and facilitators of generative AI adoption among game designers.

The qualitative analysis followed an inductive, data-driven approach. All coding was conducted by the main researcher who developed codes based on close reading of the responses and interview transcripts. Codes were then clustered into higher-level themes based on conceptual similarity and recurrence across the dataset. To enhance transparency and traceability, analytic memos were written during each phase of the coding process to document decisions, evolving interpretations, and potential connections between concepts. ATLAS.ti’s memoing and network view features were used to visualize relationships between codes, refine thematic categories, and ensure internal coherence across themes. This approach allowed for a systematic and reflexive process that captured the complexity of participants’ perspectives on generative AI.

## 4. Findings

**Figure 2:**
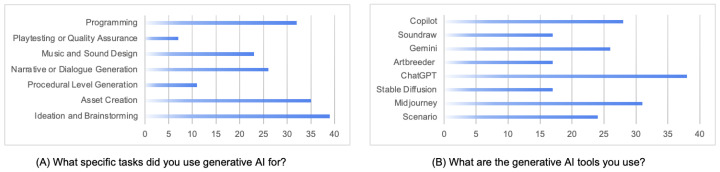
Results from survey participants highlighting generative AI usage. (**A**) Specific tasks where generative AI was applied, such as ideation and brainstorming, asset creation, and narrative/dialogue generation. (**B**) Generative AI tools frequently utilized by participants, with ChatGPT, Stable Diffusion, and Midjourney emerging as the most popular choices.

The survey results indicate how generative AI is currently being used across game design workflows (Table 1). As shown in Figure 2A, the most common application was ideation and brainstorming, reported by 90.5% of participants. This suggests that generative AI is most readily embraced in early-stage creative processes, where speed and diversity of output are especially valued. Asset creation was the second most reported use case (83.3%), indicating strong adoption of image-based tools to generate visual content such as characters, environments, or concept art. A similarly high proportion (73.8%) reported using AI for programming, which likely reflects the growing popularity of AI-assisted code generation tools.

Narrative or dialogue generation was another key area of use, cited by 66.7% of participants. This highlights the growing role of text-based generative models in prototyping in-game stories, character interactions, and branching dialogue. Less common but still notable were uses in music and sound design (47.6%) and procedural level generation (28.6%). The least frequently cited use case was playtesting or quality assurance (11.9%), suggesting that adoption in testing or gameplay balancing workflows is still limited or experimental. These trends are further supported by tool usage patterns reported in Figure 2B. The vast majority of participants (90.5%) reported using ChatGPT, which aligns with its versatility in tasks like ideation, narrative writing, and code generation. Midjourney was the second most widely used tool (76.2%), followed by Copilot (69.0%) and Gemini (64.3%). These tools reflect the diversity of generative modalities being employed—from text and images to code and system prompts.

Interestingly, tools like Scenario (61.9%) and Artbreeder (52.4%) suggest engagement with platforms that offer more stylized or customizable visual output. Fewer participants reported using Stable Diffusion (45.2%) or Soundraw (35.7%), which may reflect either lower awareness of these tools or limitations in integration with existing workflows. These tools are also viewed as an essential tool for improving efficiency and productivity within game development workflows. As one participant notes, generative AI can be utilized for “*brainstorming and ideation for proposals*” *[P3]* and “*sparking new ideas*” *[P9]* by producing quick concept art, unique level designs, and fresh character concepts. However, the perception of these tools goes beyond time-saving benefits. Game designers increasingly recognize the potential for generative AI to contribute to idea generation and creative problem-solving, pushing the boundaries of what is possible in game design. This is reflected in the survey, where over 90% of participants reported using generative AI for ideation and brainstorming tasks. These tools are viewed as collaborative partners in the creative process, offering designers unexpected solutions and new perspectives that may not emerge through traditional approaches. In interviews, designers emphasized how AI could introduce “surprising” or “novel” results that helped them break out of habitual thinking patterns and explore ideas they might not have considered on their own, framing the tools as active contributors rather than passive utilities. The role of AI, therefore, is not seen as replacing the human designer but rather augmenting human creativity by providing a powerful set of tools to explore uncharted possibilities.

Taken together, these results suggest that generative AI adoption is highest in areas where flexibility, speed, and low entry barriers align with creative needs. Tools that are intuitive, accessible, and general-purpose (like ChatGPT and Midjourney) dominate current usage, while more specialized applications (e.g., sound, QA, procedural generation) remain more niche. These results reinforce the argument that generative AI is primarily being used to augment—not replace—human-led ideation and content creation processes.

### 4.1. Perceptions of Generative AI in Game Design: Value and Potential

The insights provided by participants illustrate a paradigm shift in game design workflows, where generative AI tools are perceived as pivotal for enhancing both creative ideation and technical execution. The integration of AI into brainstorming and ideation processes reflects a growing trend in the game development industry, where designers rely on these tools to facilitate concept generation and creative exploration. As one participant noted, “*it gets me moving when I’m stuck*” *[P9]*. This suggests that AI tools are becoming essential in early-stage development, where game designers must rapidly iterate on ideas to meet project demands. The value of AI in this context lies in its ability to overcome cognitive fixation and introduce unexpected, innovative elements, thereby expanding the creative potential of human designers.

A strong majority of participants indicated high levels of openness to using generative AI in their workflows. Over 80% either agreed or strongly agreed that they intended to use generative AI in future projects, and a similarly high proportion planned to explore new tools or recommend them to others in the industry. This enthusiasm may suggest a growing interest in exploring AI-assisted practices among early adopters.

**Figure 3:**
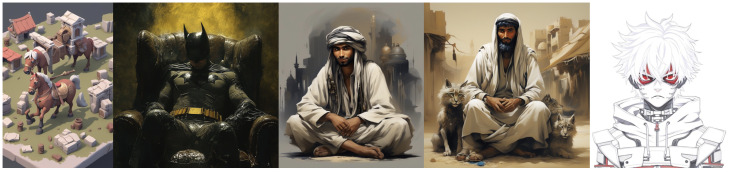
Example of game assets created by participants using generative AI tools.

In addition to augmenting creativity, generative AI tools are also recognized for their role in reducing technical barriers, particularly for independent developers. One participant mentioned that “*programming problems are now less challenging, and indie developers now have a chance to make high-quality games with less cost*” *[P6]*, which highlights the democratizing effect of generative AI in game development. Historically, small studios and independent developers faced significant resource limitations in terms of both financial investment and technical expertise. However, the adoption of AI-driven tools for procedural content generation and code optimization has lowered these barriers, enabling smaller teams to compete with larger studios. By automating tasks such as terrain generation, character creation, and asset production, generative AI allows indie developers to allocate more time to creative storytelling and gameplay mechanics, thereby increasing the overall quality of their outputs (Figure 3).

**Figure 4:**
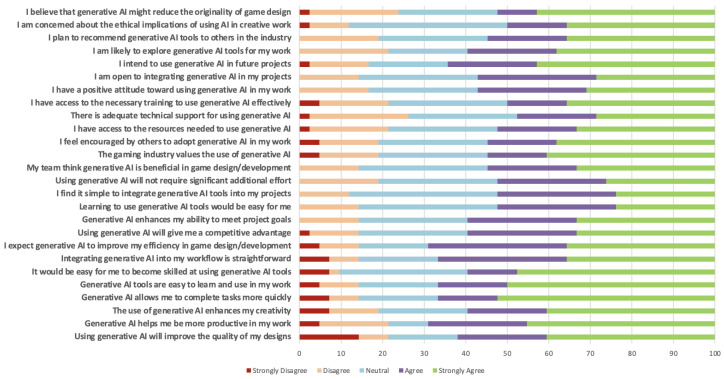
Horizontal stacked bar chart representing the combined results of TAM and UTAUT. The chart visualizes the distribution of responses across the various questions, with color-coded segments indicating the percentage of participants selecting each level of agreement.

Participants also expressed confidence in AI’s practical benefits (Figure 4). More than 75% agreed that generative AI helps them complete tasks more quickly, be more productive, and improve their design quality. These perceptions were further reinforced by strong agreement on statements like “Generative AI enhances my creativity” and “Generative AI helps me meet project goals”, suggesting that these tools are seen as efficient.

Moreover, the participants mention that they use AI for “*designing open-world environments and optimizing programming*” *[P8]*, which emphasize the dual role of AI in creative and technical processes. The creation of expansive open-world games traditionally requires extensive human input to generate dynamic landscapes, interactive elements, and non-linear narratives. Generative AI tools are now streamlining these processes by enabling automated world-building and procedural storytelling and ensuring that performance issues are addressed, and code efficiency is maintained. This intersection of creative content generation and technical problem-solving signifies a convergence of roles in game development, where designers and programmers increasingly rely on AI to collaborate across disciplines. The result is a more efficient workflow, where human creativity is augmented by AI-generated solutions that foster both innovation and practicality in game production.

### 4.2. Impact of Generative AI on Creativity, Productivity, and Efficiency

Generative AI tools are reshaping the workflows of game designers by providing novel ways to enhance creativity, productivity, and efficiency within game development environments. Participants expressed a desire for a deeper integration of generative AI capabilities within existing game engines, indicating that these tools have the potential to become integral components of game design processes. Participants suggest to “*integrate all features of generative AI tools within game engine environments*” *[P1]*, which highlights the need for seamless access to AI-driven functionalities within familiar development ecosystems. Rather than relying on external tools, participants envision a future where AI capabilities are embedded within engines such as Unity or Unreal Engine, allowing developers to streamline their workflows without switching between platforms. For example, JetBrains AI (JetBrains 2023) extends the functionality of popular IDEs by offering context-aware code suggestions, natural language explanations, and AI-assisted refactoring tools—all without leaving the development environment.

Participant largely found generative AI tools to be usable and accessible. Over two-thirds agreed that learning to use generative AI tools would be easy, and most reported finding it simple to integrate them into existing projects. This points to a relatively low barrier to adoption, especially for those already comfortable with digital design pipelines. However, some responses were more neutral when it came to training and technical support. Around one-third of participants indicate that while the generative AI tools may be intuitive, institutional or team-based infrastructure for formal learning is still developing and not widely available.

The demand for automation of repetitive or complex tasks is a recurring theme in participants’ responses. One participant noted, “*I would like game engines to add AI as a feature to do a lot of tasks like, asking it to make my object walk or give it animation based on the file animation I have added*” *[P5]*. This reflects a desire for task-specific AI assistance, where game engines can interpret commands and automatically generate animations, behaviors, or interactions based on existing assets. By reducing the time spent on manual coding and repetitive adjustments, such features would enable designers to focus more on creative problem solving and gameplay innovation. This is particularly significant for independent developers and small studios, where resources are often limited, and efficiency gains can make a substantial difference in project timelines and budgets. These patterns align with contemporary models of creative engagement in AI-supported environments, where ideation is not a linear phase but an emergent, exploratory interaction between the user and the AI system (Kinnula et al. 2024). Furthermore, the participants’ emphasis on integrated AI features suggests that generative AI has the potential to augment human creativity by removing technical barriers. Rather than viewing AI as a replacement for creative input, participants see it as a collaborative partner that can handle tedious or time-consuming tasks, thus freeing designers to explore more ambitious ideas. The integration of natural language interfaces into game engines, for example, could allow designers to issue high-level creative directives—such as generating environmental assets or character behaviors—without needing to write extensive lines of code. This enhanced efficiency could accelerate game development cycles while maintaining high-quality outputs, fostering both innovation and productivity.

This evolution from rule-based AI agents to complex generative architectures reflects a paradigm shift: AI is no longer just about making smarter NPCs or balancing difficulty curves; it is now central to rethinking what games are and how they are constructed. Instead of traditional linear pipelines, developers increasingly rely on AI-driven frameworks that integrate learning, generation, and adaptation (Gao et al. 2024a). What emerges is a symbiotic relationship between human designers and AI systems, with the latter serving as creative catalysts rather than mere tools. This trajectory points toward a new frontier in which AI techniques not only automate aspects of game development but also actively contribute to artistic direction, aesthetic coherence, and creative originality.

### 4.3. Challenges and Concerns: Barriers to Adoption of Generative AI

While generative AI tools offer significant potential to enhance efficiency and streamline workflows in game development, concerns around creativity, originality, and authorship remain critical barriers to their widespread adoption. Participants voiced apprehensions that automation might risk overshadowing human creativity, raising questions about how much of the creative process should be delegated to AI. Participants mentioned that “*Automation might risk overshadowing human creativity. Also, copyright and originality*” *[P2]*, which reflects a fundamental tension between efficiency gains provided by AI tools and the potential erosion of creative authorship. This concern is particularly relevant in an industry where originality and innovation are key to producing compelling and engaging gaming experiences.

Participants expressed hesitation in several areas, particularly around the perceived loss of creative ownership. Some raised concerns that relying on generative AI could blur the line between human and AI contributions, making it difficult to claim authorship over AI-influenced ideas. Others described situations where the AI-generated content felt generic or too similar to existing material, raising doubts about its originality. Additionally, there were concerns about how automation might reduce opportunities for junior designers, especially in roles that traditionally involve repetitive asset creation or early-stage iteration. This reflects a broader skepticism that, while AI can support efficiency, its unchecked adoption might compromise the depth of human creativity and diminish the value of manual craft in game development.

A recurring theme in participants’ responses is the fear of losing the “human touch” in game design. As one participant noted, “*A big worry is that AI might take away some of the human creativity that’s at the heart of game design. Games are often a mix of the designer’s personal vision and details. If AI handles part of the work, it might lead to a loss of the ‘human touch’ that makes a game feel unique and relatable.*” *[P7]*. These sentiments align with critiques of generative AI’s aesthetic flattening, where algorithmic outputs diminish the expressive individuality typically present in human-authored works (Kreminski 2025). Also, such concern highlight that over-reliance on AI could result in games becoming more generic or impersonal, as algorithmic outputs may lack the emotional depth and artistic nuances that human designers bring to their projects. The participant’s mention of games as a reflection of personal vision and artistic detail highlights the intimate nature of game design, where even small creative choices can have a significant impact on a player’s experience.

Additionally, there are legal and ethical concerns related to copyright and originality in the use of generative AI tools. Over 60% of participants agreed that generative AI might reduce the originality of game design, and a comparable proportion expressed concern about its ethical implications in creative work. These responses echo findings from the interviews, where participants questioned the provenance of AI-generated content and the risk of overreliance on models trained on existing creative work. The potential impact on creative authorship and labor dynamics remains a point of ambivalence. While designers recognize the value of generative tools in terms of efficiency and inspiration, they also remain cautious about how widespread adoption could affect human creativity, job roles, and the long-term cultural integrity of the games they produce.

Since AI models often rely on pre-existing datasets to generate new content, questions arise about the ownership of AI-generated assets and the potential infringement on copyrighted materials. These issues complicate the adoption of AI tools in professional workflows, particularly in large studios that must navigate intellectual property rights and legal compliance. Designers fear that the use of AI-generated content could result in unintentional plagiarism or legal disputes, making it essential for game studios to implement clear policies on how generative AI is utilized and where the boundaries of creative authorship lie.

Taken together, the findings illustrate a complex but evolving relationship between game designers and generative AI tools. While participants acknowledged substantial benefits—particularly in ideation, prototyping, and workflow acceleration—they also voiced concerns related to creative autonomy, authorship, and ethical uncertainty. These tensions set the stage for a broader discussion about how generative AI is reshaping professional practices, team dynamics, and cultural expectations within game design.

## 5. Discussion

The results of this study reveal a complex and multifaceted relationship between game designers and generative AI tools, highlighting both their growing influence and the challenges they present. The overarching sentiment among participants reflects a cautiously optimistic embrace of these tools, with a clear recognition of their ability to enhance productivity, creativity, and efficiency. At the same time, critical concerns about originality, ethics, and resource accessibility highlight the need for a nuanced approach to their integration in game design workflows. One of the insights from the data is the duality of perception surrounding creativity. On one hand, generative AI is widely regarded as a catalyst for ideation and exploration, enabling designers to experiment with novel concepts and iterate quickly. This aligns with its perceived role as a tool that complements human ingenuity, enhancing the creative process rather than replacing it. Yet, there remains a degree of skepticism about the potential homogenization of creative outputs. Over a third of participants expressed concern that generative AI might undermine originality, raising important questions about whether reliance on algorithmic generation could unintentionally diminish the authenticity of game designs. This tension highlights that, while generative AI accelerates the creative process, it simultaneously compels designers to safeguard their artistic vision in the face of increasingly convenient automated solutions.

These findings resonate with current research about AI as a co-creative agent. Prior research suggest that generative systems shift the creative process from authorship to curation, a pattern echoed by participants who described filtering and refining AI-generated outputs. This aligns with theories of distributed creativity, where human and machine agency jointly shape the design outcome (Li 2024; Panchanadikar and Freeman 2024). Moreover, the ethical concerns raised by participants—including attribution, originality, and labor displacement—mirror broader debates in the literature on responsible AI integration in creative industries (Singh et al. 2024). Our findings reinforce the need for transparent AI systems that support human intent, echoing calls for ethical frameworks that maintain clarity of authorship, protect creative labor, and ensure accountability in AI-assisted production.

Another critical insight lies in the accessibility and usability of generative AI tools. Participants generally viewed these tools as intuitive and straightforward to integrate into their workflows, reflecting their maturity and user-centric design. However, the data also reveal gaps in training and resource availability, with a significant minority indicating they lacked the necessary support to fully leverage these tools. This points to an opportunity for the industry to invest in training programs and technical resources to democratize access to generative AI, ensuring that its benefits are not limited to those with prior expertise or access to privileged resources.

### 5.1. Disruption to Traditional Design Workflows and Tools

Participants drew comparisons between generative AI tools and traditional design workflows and tools, revealing a complex mix of enthusiasm and skepticism. Many acknowledged that generative AI tools, particularly for image and asset generation, significantly accelerate early-stage ideation. Concept art that might have taken hours to draft manually could now be created in minutes using tools like Midjourney or DALL·E (Naik et al. 2024). However, this speed was often described as coming at the expense of control, precision, or intentional artistic expression. Designers noted that, while AI-generated visuals are compelling, they often lack the deliberate composition or stylistic nuance associated with hand-drawn sketches, making them less suitable for finalized assets without further human refinement.

Several participants framed generative AI as a natural extension to existing workflows, particularly in areas like concept development, asset prototyping, and narrative drafting. Rather than viewing these tools as disruptive replacements, many saw them as augmentative technologies that could slot into familiar stages of the design pipeline. For example, designers described using AI-generated outputs as a starting point—such as rough concept art or dialogue drafts—which were then refined through traditional tools and human judgment (Lee et al. 2023). This framing reflects a pragmatic orientation: generative AI is not positioned as a wholesale transformation, but as a complementary layer that enhances speed and ideation within already established processes. These reflections offer early insights into how designers evaluate the trade-offs between established creative methods and AI-driven innovation. Rather than replacing traditional workflows, generative AI appears to supplement them—speeding up certain phases while complicating others. The challenge, as noted by several designers, lies in finding a balanced integration strategy that preserves artistic control while leveraging the generative potential of the tools.

Participants also described generative AI as a “creative companion” that supports different phases of the design pipeline (Zaccolo 2020). In the ideation stage, tools like ChatGPT and Midjourney are used to overcome creative blocks and spark novel directions by generating mood boards, concept sketches, or sample prompts. During asset development, AI tools help accelerate prototyping by producing placeholder visuals or draft dialogues that can be iteratively refined. It can fill the gap between the rough idea and the first playable version, allowing for rapid exploration without delaying production timelines. In later stages, such as narrative refinement or level balancing, AI-generated content often serves as a reference point for human iteration, prompting teams to make strategic decisions about what to keep, alter, or discard. This layered interaction reflects a fluid partnership in which AI is not just a task automator but a co-creative element shaping the trajectory of the design process.

### 5.2. Ethical Implications of Generative AI

The ethical implications of generative AI also emerged as a recurring theme. While the tools are praised for their ability to automate repetitive tasks and enhance efficiency, there is a palpable concern about their impact on the integrity of human-authored content (Naik et al. 2024; Singh et al. 2024). Designers expressed unease about the potential erosion of originality and the risk of over-reliance on AI-generated assets. These concerns highlight a broader tension between the technological advancement AI represents and the creative autonomy that defines the identity of game designers. To address this, there is a pressing need to develop ethical guidelines that promote responsible use of generative AI, ensuring that its contributions augment rather than overshadow human creativity.

A significant point of concern was whether content produced by generative models can truly reflect a distinct creative vision. Participants questioned the intentionality and expressive depth of AI-generated outputs, describing them as often lacking the nuance, emotional tone, and stylistic coherence that characterize human-made work. These concerns were not limited to aesthetic judgments; they pointed to a broader anxiety about the dilution of authorship and the shifting boundaries of creative ownership in collaborative projects. Similar critiques have emerged in other design fields such as architecture, where the use of generative models has been associated with stylistic uniformity and reduced expressive range (Kudless 2024).

Several participants also raised issues regarding the provenance of AI-generated content, particularly when models are trained on copyrighted or unattributed material. This prompted ethical questions about legitimacy, originality, and accountability. The opacity of these systems, combined with their reliance on vast datasets, fueled skepticism about the supposed neutrality of generative AI. Participants called for greater transparency in model training processes and clearer attribution practices in collaborative settings. A recurring theme among participants was the ambiguity surrounding the ownership of AI-generated content. Designers voiced uncertainty about whether assets created with generative tools—especially those trained on publicly scraped or copyrighted data—could be safely used in commercial projects. This concern was particularly relevant for visual and narrative assets, where stylistic overlap or unintended replication of existing works could pose legal risks. Without clear documentation of training data sources or licensing terms, some participants reported being hesitant to integrate AI-generated outputs directly into final products. Instead, they opted to use these assets strictly for prototyping or internal ideation, avoiding distribution or publication.

Importantly, these ethical concerns extended into the domain of professional risk. Multiple participants mentioned that the normalization of generative AI could lead to workforce reductions, particularly affecting junior artists, writers, and designers. In smaller studios with tight budgets, AI was seen not just as a tool to support human labor but as a potential replacement for it. This introduces a labor ethics dimension to the discussion, where creative integrity intersects with concerns about job security, fair compensation, and the long-term sustainability of creative professions.

While some participants expressed concern about the potential for AI to displace human creativity, other scholars present a contrasting view—arguing that generative AI democratizes access to creative expression. For instance, by lowering technical and artistic entry barriers, AI tools enable individuals with limited formal training in art or programming to contribute meaningfully to game design. This perspective aligns with literature that sees generative AI as an enabler of broader participation in creative industries, empowering smaller teams or solo developers to prototype and produce content at levels previously restricted to larger studios (Lee et al. 2023; Panchanadikar and Freeman 2024). Our findings partially support this view, with several participants noting that AI allowed them to “do more with less” or take on roles they would otherwise need to outsource.

Taken together, these findings suggest that ethical ambivalence toward generative AI is not rooted in technological conservatism, but in a desire to protect the visibility of human authorship, ensure equitable labor practices, and uphold the cultural and professional standards of game design (Boucher et al. 2024; Singh et al. 2024). As generative tools become more embedded in creative workflows, these concerns will require not only technical and policy-based solutions, but also critical dialogue across the industry about the values that should guide AI integration in creative work.

### 5.3. Playful Interaction

Participants in this study described moments of playful experimentation with generative AI tools, especially during ideation and early-stage design. Several participants noted that they would set parameters or input prompts and then step back to see what the AI produced, engaging in what can be described as “playing” with the tool (Chien et al. 2024; Lee et al. 2025; Matthews et al. 2024). This way of interacting—taking turns between giving input and observing results—suggests that generative AI can act like a creative partner, where the designer is not just controlling the tool but also reacting to it. Instead of following a fixed plan, participants treated such tools as something to explore, test, and even be surprised by. Some shared that unexpected outputs led them to new ideas or helped them move past creative blocks. These back-and-forth moments between the user and the tool are similar to how players interact in *idle games*—games that progress without player interaction for some time—where both sides, the player and the game, contribute to the final result through small, ongoing steps (Alharthi et al. 2018; Spiel et al. 2019). This playful way of working with AI shows that design using generative AI tools isn’t just about solving problems quickly, but also about staying open to unexpected outcomes and creative and playful exploration.

### 5.4. Generative AI Adoption in Collaborative Work

Equally important is the social and collaborative dimension of generative AI adoption. Participants expressed a strong willingness to recommend and explore these tools within their professional networks, suggesting a growing sense of acceptance and enthusiasm. However, this enthusiasm is tempered by the need for clear guidance on how to integrate AI seamlessly into collaborative workflows. The results indicate that fostering a culture of experimentation and knowledge sharing within teams could help alleviate apprehensions and enable designers to harness the full potential of these tools.

Furthermore, reusing certain prompt structures or interaction habits mirrors the concept of *game design patterns* (Bjork and Holopainen 2005; Toups et al. 2016), suggesting that co-creating with generative AI may give rise to its own repertoire of repeatable practices—or *prompt engineering patterns*—that function as creative templates over time. However, the absence of clear standards or protocols for using AI-generated content in collaborative settings emerged as a consistent theme. Participants raised concerns about attribution—such as whether AI-generated content should be credited differently—as well as expectations for human refinement and the need for shared conventions to label or verify AI-assisted contributions. These ambiguities were seen as potential barriers to trust and transparency within collaborative environments. These challenges echo broader concerns in cooperative digital design settings. Prior research highlight the importance of shared awareness in creative collaboration—even when the collaboration is between human peers rather than with AI (Alharthi et al. 2022).

Overall, the findings highlight the significant potential of generative AI in game design, but they also highlight the critical need for intentionality in its adoption. Designers and developers must focus on finding a balance between leveraging the speed and efficiency AI offers and safeguarding the core principles of originality, ethics, and human creativity. Future efforts should focus on developing collaborative frameworks that position AI as a creative partner rather than a substitute, fostering an environment where technology amplifies rather than diminishes the unique contributions of human designers. By addressing these challenges, the game design and development industry can explore the evolving potential of generative AI as a tool for innovation, collaboration, and creativity.

## 6. Limitations

While this study provides valuable insights into the adoption and impact of generative AI in game design, several limitations should be acknowledged. The study relied on a relatively small sample of game designers and developers from the MENA region, which may limit the statistical power and generalizability of the findings across the entire game design industry. While efforts were made to ensure diversity in participants’ roles, experience levels, and project scopes, the sample may not fully represent the broader game development landscape and are not statistically generalizable but aim to uncover emerging themes and perspectives among practitioners. Additionally, participants were recruited from a game design and development social media community, which may introduce selection bias. Those who chose to participate may have a particular interest in generative AI, potentially skewing the results toward more favorable or critical views of generative AI.

The study also relies on self-reported data from surveys and interviews, which may be subject to response biases. While qualitative insights provide depth, they reflect individual perceptions and experiences rather than objective measures of AI’s impact on game design processes. Although established coding procedures were followed for thematic analysis (Braun and Clarke 2012), the coding was performed by a single researcher. Consequently, interpretations reflect an individual perspective. Other researchers may identify different themes or interpret the themes from this study differently. Further research utilizing observational or experimental approaches could help validate these findings. Additionally, while the study examines the use of generative AI, it does not extensively compare AI tools with traditional game design methods. A more comprehensive comparative analysis would provide additional context regarding the advantages and limitations of AI relative to conventional creative workflows.

Although our sample included participants from both independent and studio-based backgrounds, the study was not structured to explicitly compare experiences across different organizational contexts. As a result, we can not make strong claims about how factors such as team size, funding, or institutional infrastructure influence the adoption of generative AI. Future studies may benefit from segmenting participants by studio scale to better understand how resource availability shape perceptions of AI tools.

Furthermore, the study touches on ethical concerns, such as authorship, originality, and the potential displacement of creative professionals, but does not provide an exhaustive analysis of these topics. Given the evolving nature of AI ethics, future research should further investigate how stakeholders navigate these challenges and how emerging policies might shape AI’s role in game design. Despite these limitations, the findings contribute valuable insights into the growing role of generative AI in game design and development and provide a foundation for further exploration in this rapidly evolving field.

## 7. Conclusions

This study examined how generative AI tools are shaping creativity and innovation in game design and development. Our findings highlight a dual reality: while these tools enhance ideation, accelerate prototyping, and reduce manual workload, they also raise concerns about originality, authorship, and ethical use of AI-generated content. These tensions highlight the need for thoughtful integration strategies that balance efficiency with creative integrity. To support this balance, game designers and developers should establish clear internal protocols—defining when and how AI-generated content is used, ensuring proper attribution, and maintaining human oversight throughout the design process. Educators must equip future designers and developers with both technical fluency and ethical awareness, preparing them to critically engage with AI tools in professional environments. Policymakers, in turn, must introduce regulatory frameworks that clarify ownership, protect creative labor, and ensure transparency around the provenance of AI-generated assets. The urgency of regulation is particularly pressing as generative AI becomes more embedded in creative workflows. Without timely and clear guidance, developers risk navigating legal uncertainty and ethical ambiguity, particularly around intellectual property. Practical interventions at the organizational, educational, and policy levels are essential to ensure that generative AI supports—rather than undermines—the values of authorship, equity, and creative autonomy in game design and development.

## Figures and Tables

**Table 1:** Summary of the main themes emerged from the thematic analysis.

Theme	Description
Enhanced Ideation	Generative AI tools are widely employed during the conceptual stages of design to facilitate brainstorming, overcome creative blocks, and inspire novel concepts. Designers find these tools particularly useful in rapidly generating diverse ideas.
Prototyping and Efficiency	AI significantly enhances productivity by automating time-intensive tasks such as asset creation, scripting, and early-stage prototyping. This leads to faster iteration cycles and allows developers to focus more on creative refinement.
Creative Augmentation	Rather than viewing AI as a replacement, designers describe it as a collaborative agent that amplifies human creativity. AI is valued for its ability to generate surprising, idea-provoking content that expands the creative process.
Concerns about Originality	Participants express apprehension that AI-generated content may be unoriginal or overly reliant on training data, resulting in derivative outputs. These concerns center around preserving the authenticity and uniqueness of human-led design.
Ethical and Legal Ambiguity	Uncertainty surrounds the attribution and copyright status of AI-generated content. Designers raise questions about the ethical legitimacy and legal risks of using material derived from opaque training datasets.
Democratization	Generative AI lowers barriers to entry by offering accessible tools that reduce the need for specialized skills. Independent developers and small teams benefit from AI’s ability to streamline tasks that would otherwise require extensive resources.
Labor Displacement	While AI enhances efficiency, it also raises fears of reducing opportunities for junior designers and artists. Automation of foundational creative tasks may shift employment dynamics within the game development ecosystem.
Collaborative Workflow	Integration of AI-generated content into team-based projects remains a challenge. Issues such as authorship attribution, transparency, and shared conventions for crediting AI outputs were frequently cited as barriers to effective collaboration.
Usability and Integration	Tools like ChatGPT and Midjourney are celebrated for their ease of use and versatility, yet participants noted limitations in adapting these tools to specific workflows, such as procedural generation or engine integration.
Efficiency and Human Touch	Designers emphasized the need to strike a balance between the speed, scalability, and automation afforded by generative AI and the emotional depth, stylistic coherence, and personal expression that define compelling and meaningful game experiences.

## Data Availability

The original contributions presented in this study are included in the article. Further inquiries can be directed to the corresponding author.

## References

[B1-jintelligence-13-00060] Alharthi Sultan A., Olaa Alsaedi, Toups Dugas Phoebe O., Jean Tanenbaum Theresa, Jessica Hammer (2018). Playing to Wait: A Taxonomy of Idle Games. Paper presented at the 2018 CHI Conference on Human Factors in Computing Systems.

[B2-jintelligence-13-00060] Alharthi Sultan A., Ben Lafreniere, Tovi Grossman, George Fitzmaurice (2022). TwoTorials: A Remote Cooperative Tutorial System for 3D Design Software In *Graphics Interface 2022*. https://openreview.net/forum?id=H2GICxFVaGc.

[B3-jintelligence-13-00060] Amresh Ashish (2023). Leveling up education: Harnessing generative AI for game-based learning. Paper presented at the 16th Annual ACM India Compute Conference.

[B4-jintelligence-13-00060] Arnecke Jörn, Eck Sebastian Oliver, Steuck Pia, Vaughan Alexander (2024). Integrating generative AI in music education: With ai in a musical question-answer game. Paper presented at the INFORMATIK 2024.

[B5-jintelligence-13-00060] Bao Aorigele, Zeng Yi (2024). Allowing AI co–authors is a disregard for humanization. Accountability in Research.

[B6-jintelligence-13-00060] Begemann Andrew, Hutson James (2024). Empirical insights into AI-assisted game development: A case study on the integration of generative ai tools in creative pipelines. Metaverse.

[B7-jintelligence-13-00060] Björk Staffan, Holopainen Jussi (2005). Games and design patterns. The Game Design Reader: A Rules of Play Anthology.

[B8-jintelligence-13-00060] Boucher Josiah D., Smith Gillian, Telliel Yunus Doğan (2024). Is resistance futile?: Early career game developers, generative AI, and ethical skepticism. Paper presented at CHI Conference on Human Factors in Computing Systems.

[B9-jintelligence-13-00060] Boucher Josiah D., Smith Gillian, Telliel Yunus (2023). Examining early professionals’ use of generative AI in the game development process. EXAG@ AIIDE.

[B10-jintelligence-13-00060] Braun Virginia, Clarke Victoria (2012). Thematic Analysis.

[B11-jintelligence-13-00060] Chen Ke, Vinjamuri Ramana, Wang Honggang, Kadiyala Sai Praveen (2024). Generative AI based difficulty level design of serious games for stroke rehabilitation. IEEE Internet of Things Journal.

[B12-jintelligence-13-00060] Chien Chih-Chung, Chan Hung-Yu, Hou Huei-Tse (2024). Learning by playing with generative AI: Design and evaluation of a role-playing educational game with generative AI as scaffolding for instant feedback interaction. Journal of Research on Technology in Education.

[B13-jintelligence-13-00060] Choi Bu-Ho (2024). Investigating learner perceptions for effective teaching of generative AI-from a game development perspective. Journal of The Korea Society of Computer and Information.

[B14-jintelligence-13-00060] Colado Iván J. Pérez, Colado Víctor M. Pérez, Morata Antonio Calvo, Píriz Rubén Santa Cruz, Manjón Baltasar Fernández (2023). Using new AI-driven techniques to ease serious games authoring. Paper presented at the 2023 IEEE Frontiers in Education Conference (FIE).

[B15-jintelligence-13-00060] Davis Fred D., Bagozzi Richard P., Warshaw Paul R. (1989). Technology acceptance model. Journal of Management and Science.

[B16-jintelligence-13-00060] Ersoy Berkin (2024). AI AT PLAY: Exploratory Study on the Drivers Behind Generative AI Adoption for Game Art Asset Production. Master’s thesis.

[B17-jintelligence-13-00060] Gao Fengsen, Fang Ke, Chan Wai Kin (2024a). Chemate: Anthropomorphic-cues-mediated experiential learning game using generative AI. Paper presented at the International Conference on Human-Computer Interaction.

[B18-jintelligence-13-00060] Gao Fengsen, Fang Ke, Chan Wai Kin (2024b). Humanizing artifacts: An educational game for cultural heritage artifacts and history using generative ai. Paper presented at the Companion Proceedings of the 2024 Annual Symposium on Computer-Human Interaction in Play.

[B19-jintelligence-13-00060] Giray Louie (2023). Prompt engineering with ChatGPT: A guide for academic writers. Annals of Biomedical Engineering.

[B20-jintelligence-13-00060] Hendrikx Mark, Meijer Sander, Velden Jeroen Van Der, Iosup Alexandru (2013). Procedural content generation for games: A survey. ACM Transactions on Multimedia Computing, Communications, and Applications (TOMM).

[B21-jintelligence-13-00060] Hutson James, Fulcher Ben, Jeremiah Ratican (2024). Enhancing assessment and feedback in game design. International Journal of Educational Research and Innovation.

[B22-jintelligence-13-00060] JetBrains (2023). JetBrains AI Assistant. https://www.jetbrains.com/ai/.

[B23-jintelligence-13-00060] Khalifa Ahmed, Bontrager Philip, Togelius Julian, Nielsen Jonny (2018). Talakat: Bullet hell generation through constrained map-elites. Paper presented at the 2018 IEEE Conference on Computational Intelligence and Games (CIG).

[B24-jintelligence-13-00060] Kim Munyeong, Kim Sungsu (2023). Generative AI in mafia-like game simulation. arXiv.

[B25-jintelligence-13-00060] Kinnula Marianne Eva Durall Gazulla, Hirvonen Noora, Malmberg Jonna, Haukipuro Lotta (2024). Nurturing systems thinking among young people by developing business ideas on sustainable AI. International Journal of Child-Computer Interaction.

[B26-jintelligence-13-00060] Kreminski Max (2025). Endless forms most similar: The death of the author in AI-supported art. AI & Society.

[B27-jintelligence-13-00060] Kudless Andrew (2024). Stop Making Sense: Complexity and Contradiction in AI and Architecture. Architectural Design.

[B28-jintelligence-13-00060] Lee Cassandra, Dimitra Dimitrakopoulou, Deb Roy (2025). Meeting at Crossroads: An exploration of playful listening through a co-creative AI game. Paper presented at the Extended Abstracts of the CHI Conference on Human Factors in Computing Systems.

[B29-jintelligence-13-00060] Lee JaeJun, Eom So-Youn, Lee JunHee (2023). Empowering game designers with generative AI. IADIS International Journal on Computer Science & Information Systems.

[B30-jintelligence-13-00060] Li Cheng-Tai, Lee Liang-Hsuan, Hou Huei-Tse (2024). An educational simulation game integrated with generative AI as conversational contextual scaffolding for business english: A preliminary analysis of learning achievement, self-efficacy and cognitive load. Paper presented at the 2024 16th IIAI International Congress on Advanced Applied Informatics (IIAI-AAI).

[B31-jintelligence-13-00060] Li Shaojun (2024). Using Generative AI for Teaching Game Development Using Unity. Master’s thesis.

[B32-jintelligence-13-00060] Liang Xiaozhan, Wang Yu, Yan Fengyi, Ouyang Zehong, Hu Yong, Luo Siyu (2024). Reborn of the white bone demon: Role-playing game design using generative AI in XR. Paper presented at the SIGGRAPH Asia 2024 Posters.

[B33-jintelligence-13-00060] Liapis Antonios, Yannakakis Georgios N., Togelius Julian (2013). Sentient sketchbook: Computer-assisted game level authoring. Paper presented at the 8th International Conference on the Foundations of Digital Games.

[B34-jintelligence-13-00060] Maleki Mahdi Farrokhi, Zhao Richard (2024). Procedural content generation in games: A survey with insights on emerging llm integration. Paper presented at the AAAI Conference on Artificial Intelligence and Interactive Digital Entertainment.

[B35-jintelligence-13-00060] Matthews Jenna, Nguyen Ha, Swanson Hillary (2024). Shall we play a game? distributed games with a generative AI player. Paper presented at the 17th International Conference on Computer-Supported Collaborative Learning-CSCL 2024.

[B36-jintelligence-13-00060] Medeiros Kelsey, Cropley David H., Marrone Rebecca L., Reiter-Palmon Roni (2025). Human-AI Co-Creativity: Does ChatGPT Make Us More Creative?. The Journal of Creative Behavior.

[B37-jintelligence-13-00060] Muengsan Suthada, Chatwattana Pinanta (2024). The game-based learning (gbl) platform with generative ai to enhance digital and technology literacy skills. Higher Education Studies.

[B38-jintelligence-13-00060] Naik Tanvi, Hrishikumar Gostu, Rahul Sharma (2024). Navigating Ethics of AI-Powered Creativity in Midjourney. Paper presented at the 3rd International Conference for Innovation in Technology.

[B39-jintelligence-13-00060] Panchanadikar Ruchi, Freeman Guo (2024). “I’m a solo developer but AI is my new ill-informed co-worker”: Envisioning and designing generative AI to support indie game development. Proceedings of the ACM on Human-Computer Interaction.

[B40-jintelligence-13-00060] Panchanadikar Ruchi, Freeman Guo, Li Lingyuan, Schulenberg Kelsea, Hu Yang (2024). “A new golden era” or “slap comps”: How non-profit driven indie game developers perceive the emerging role of generative AI in game development. Paper presented at the Extended Abstracts of the CHI Conference on Human Factors in Computing Systems.

[B41-jintelligence-13-00060] Pérez-Colado Iván J., Freire-Morán Manuel, Calvo-Morata Antonio, Pérez-Colado Víctor M., Fernández-Manjón Baltasar (2024). AI asyet another tool in undergraduate student projects: Preliminary results. Paper presented at the 2024 IEEE Global Engineering Education Conference.

[B42-jintelligence-13-00060] Qiu Shanhui (2023). Generative AI processes for 2d platformer game character design and animation. Lecture Notes in Education Psychology and Public Media.

[B43-jintelligence-13-00060] Ranella Noah, Eger Markus (2023). Towards automated video game commentary using generative AI. EXAG@ AIIDE.

[B44-jintelligence-13-00060] Ratican Jay, Hutson James (2024). Adaptive worlds: Generative AI in game design and future of gaming, and interactive media. ISRG Journal of Arts, Humanities and Social Sciences.

[B45-jintelligence-13-00060] Shan Tiger, Michel Kay (2024). Generative AI with goap for fast-paced dynamic decision-making in game environments. Paper presented at the 2024 IEEE Conference on Games (CoG).

[B46-jintelligence-13-00060] Singh Gulbir, Srivastava Vivek, Kumar Suneet, Bhatnagar Vivek, Dhondiyal Shiv Ashish (2024). Understanding the ethical implications of generative AI: A multi-disciplinary perspective. Paper presented at the 2024 5th International Conference on Electronics and Sustainable Communication Systems (ICESC).

[B47-jintelligence-13-00060] Smith Gillian, Whitehead Jim, Mateas Michael (2010). Tanagra: A mixed-initiative level design tool. Paper presented at the Fifth International Conference on the Foundations of Digital Games.

[B48-jintelligence-13-00060] Spiel Katta, Alharthi Sultan A., Jian-lan Cen Andrew, Jessica Hammer, Nacke Lennart E., Toups Dugas Phoebe O., Jean Tanenbaum Theresa (2019). “It Started as a Joke”: On the Design of Idle Games. Paper presented at the Annual Symposium on Computer-Human Interaction in Play.

[B49-jintelligence-13-00060] Sternberg Robert J. (2024). Do not worry that generative AI may compromise human creativity or intelligence in the future: It already has. Journal of Intelligence.

[B50-jintelligence-13-00060] Sun Bo Cun, Cho Dong Min (2024). A study on the creativity of creators using genai tools in game ugc—Focusing on roblox. The Korean Society of Science & Art.

[B51-jintelligence-13-00060] Sun Yuqian, Li Zhouyi, Fang Ke, Lee Chang Hee, Asadipour Ali (2023). Language as reality: A co-creative storytelling game experience in 1001 nights using generative AI. Paper presented at the AAAI Conference on AI and Interactive Digital Entertainment.

[B52-jintelligence-13-00060] Sweetser Penny (2024). Large language models and video games: A preliminary scoping review. Paper presented at the 6th ACM Conference on Conversational User Interfaces.

[B53-jintelligence-13-00060] Toups Dugas Phoebe O., Hamilton William A., Alharthi Sultan A. (2016). Playing at Planning: Game Design Patterns from Disaster Response Practice. Paper presented at the 2016 Annual Symposium on Computer-Human Interaction in Play.

[B54-jintelligence-13-00060] Ubisoft La Forge (2024). Ubisoft La Forge—Bridging the Gap Between Academic Research and Video Game Innovation. https://www.ubisoft.com/en-us/studio/laforge.

[B55-jintelligence-13-00060] Venkatesh Viswanath, Thong James YL, Xu Xin (2012). Consumer acceptance and use of information technology: Extending the unified theory of acceptance and use of technology. MIS Quarterly.

[B56-jintelligence-13-00060] Vinchon Florent, Gironnay Valentin, Lubart Todd (2024). Genai creativity in narrative tasks: Exploring new forms of creativity. Journal of Intelligence.

[B57-jintelligence-13-00060] Wang Qingyang (2024). Generative AI in Game Pedagogy. Ph.D. thesis.

[B58-jintelligence-13-00060] Watanabe Neo, Kano Yoshinobu (2024). Werewolf game agent by generative AI incorporating logical information between players. Paper presented at the 2nd International AIWolfDial Workshop.

[B59-jintelligence-13-00060] Werning Stefan (2024). Generative AI and the technological imaginary of game design. Creative Tools and the Softwarization of Cultural Production.

[B60-jintelligence-13-00060] Whitaker Elizabeth, Trewhitt Ethan, Veinott Elizabeth (2024). Heuristica II: Updating a 2011 game-based training architecture using generative AI tools. Paper presented at the 6th International Conference on Human-Computer Interaction.

[B61-jintelligence-13-00060] Yannakakis Georgios N., Togelius Julian (2018). Artificial Intelligence and Games.

[B62-jintelligence-13-00060] Zaccolo Sandro (2020). Artificial Intelligence as a Creativity Companion.

